# Creation of bioactive non‐natural flavonoids via combinatorial prenylation–glycosylation cascades

**DOI:** 10.1002/mlf2.70063

**Published:** 2026-03-15

**Authors:** Hongjiao Zhang, Anxin Zhang, Jiatong Ji, Wen‐Bing Yin

**Affiliations:** ^1^ State Key Laboratory of Microbial Diversity and Innovative Utilization, Institute of Microbiology, Chinese Academy of Sciences Beijing China; ^2^ Medical School University of Chinese Academy of Sciences Beijing China; ^3^ Division of Life Sciences and Medicine University of Science and Technology of China Hefei China

## Abstract

Flavonoids have significant medicinal potential; however, the reliance on plant extraction for their production poses a substantial barrier to their practical application. Innovative approaches like enzymatic cascade catalysis and microbial synthesis present promising avenues for the efficient production of these structurally complex compounds. In this study, two non‐natural flavonoids (compounds **7** and **8**) were synthesized, with compound **7** displaying antifungal properties. This was achieved through the cascade enzymatic catalysis of naringenin, a key intermediate in flavonoid biosynthesis, using glycosyl‐ and prenyltransferases. Additionally, metabolic engineering was used to bolster the supply of the dimethylallyl pyrophosphate precursor via the isopentenol utilization pathway in *Escherichia coli*, facilitating the *de novo* creation of non‐natural flavonoids. Consequently, the titers of compounds **7** and **8** reached 47.4 and 6.6 mg/l, respectively. This research highlights the potential of modular enzyme assembly in generating bioactive flavonoid derivatives and establishes a sustainable platform for the discovery of non‐natural flavonoids.

Flavonoids are one of the largest and most widely distributed classes of polyphenolic compounds in the plant kingdom, showing a broad spectrum of biological activities that are beneficial to human health^1^, ^2^, such as antioxidant, anti‐inflammatory, antimicrobial, and anticancer effects. These natural products are characterized by a 15‐carbon skeleton arranged in a C6–C3–C6 pattern and are subdivided into several major classes, including flavones, flavonols, flavanones, and isoflavonoids^3^. Despite their limited core structural diversity, the complexity of flavonoids arises from extensive post‐synthetic modifications^4^ including glycosylation, prenylation, methylation^5^, hydroxylation, and acetylation. These modifications enhance their bioavailability. Naringenin, a flavanone predominantly found in citrus fruits, is a key biosynthetic intermediate and has various documented bioactivities^6^. However, the application of naringenin and many other flavonoids is often constrained by their relatively low bioavailability, poor water solubility, and insufficient structural diversity.

To overcome these limitations, structural modification through enzymatic glycosylation^7^ and prenylation^8^ has emerged as a powerful strategy. Glycosylation, typically catalyzed by UDP‐dependent glycosyltransferases (UGTs), enhances water solubility and stability by introducing sugar moieties to hydroxyl groups. Prenylation, mediated by prenyltransferases (PTs), attaches lipophilic prenyl groups (e.g., dimethylallyl diphosphate derivatives) to the flavonoid skeleton, significantly increasing membrane permeability and often enhancing biological activity^9^. Naturally occurring prenylated and glycosylated flavonoids, such as 8‐prenylnaringenin and its glucosides, demonstrate improved pharmacological profiles but are typically produced in low quantities and are challenging to extract and purify. Consequently, enzymatic biosynthesis has emerged as an eco‐friendly production strategy, such as the production of vitexin^10^ with a titer of 935.6 mg/l in a one‐pot enzymatic cascade from naringenin and kaempferol, with a yield of 37.55 mg/l in an *in vitro* multienzyme synthetic system^11^.

Advances in synthetic biology and enzyme engineering now enable the design and production of “non‐natural” flavonoid derivatives through combinatorial biosynthesis^12^. By harnessing the versatility of tailoring enzymes from various biological origins, it is feasible to synthesize novel compounds with improved properties. In this study, we documented the effective biosynthesis of two new naringenin derivatives, achieved by combining a bacterial glycosyltransferase with a fungal prenyltransferase, both *in vitro* and within genetically engineered *Escherichia coli*. Additionally, we showcase the antifungal efficacy of one of these new compounds against *Aspergillus niger*, underscoring the potential of this method in generating novel bioactive molecules.

In this study, we chose naringenin (**1**) as the core substrate for enzymatic modification due to its central role in flavonoid biosynthesis and its well‐established bioactivities. To create structurally novel derivatives, we evaluated various glycosyltransferases (UGT73C6^13^ from *Arabidopsis thaliana*, CsUGT75L12^14^ from *Camellia sinensis*, CiUGT11^15^ from *Chrysanthemum indicum*, UGT73B1^16^ from *A. thaliana*, and YjiC^17^ from *Bacillus licheniformis*) and prenyltransferases (7‐DMATS^18^ from *Aspergillus fumigatus* and AnaPT^19^ from *Neosartorya fischeri*) for their activity on naringenin and compatibility in cascade reactions (Table S1). An *in vitro* enzymatic reaction demonstrated that YjiC showed product diversity among the tested UGTs, producing naringenin 4′,7‐*O*‐diglucoside (**2**), 7‐*O*‐glucoside (**3**), and 4′‐*O*‐glucoside (**4**) as products (Figures 1A and S1). For prenylation, the fungal prenyltransferase AnaPT was selected due to its high efficiency in transferring a prenyl group to the C‐3′ position of naringenin (**5**), a relatively uncommon modification that provided novelty in our design, and to the C‐6 position of naringenin (**6**) (Figure 1B).

**Figure 1:**
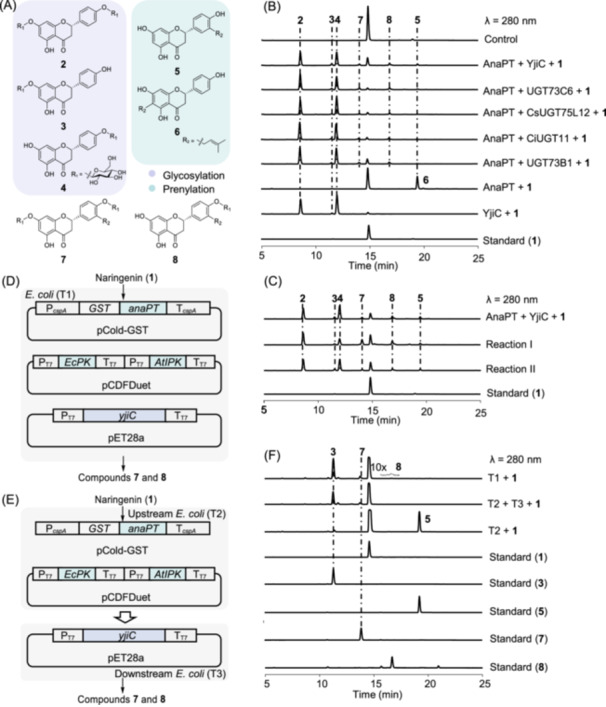
Creation of novel prenylated and glycosylated naringenin derivatives via combinatorial biosynthesis. (A) Chemical structures of naringenin modified by glycosyltransferases and prenyltransferases: **2**, naringenin 4′,7‐*O*‐diglucoside; **3**, naringenin 7‐*O*‐glucoside; **4**, naringenin 4′‐*O*‐glucoside; **5**, 3′‐prenylnaringenin (3′‐PN); and **6**, 6‐prenylnaringenin (6‐PN). (B) High‐Performance Liquid Chromatography (HPLC) analysis of the enzymatic reaction involving five UGTs and AnaPT with substrate **1**. (C) HPLC analysis of the enzymatic reaction in two reaction systems. Reaction I: AnaPT was incubated with naringenin and DMAPP for 2 h at 37°C, followed by the immediate addition of YjiC and UDP‐glucose for another 2 h at 37°C. Reaction II was conducted in the reverse order. (D) Schematic diagram of a mono‐culture experiment using the *Escherichia coli* BL21 strain expressing the *anaPT*, *yjiC*, and IUP (*EcPK* and *AtIPK*) genes. (E) Schematic diagram of a co‐culture experiment involving an *E. coli* strain expressing the *anaPT* and IUP genes and another *E. coli* strain expressing *yjiC*. (F) HPLC analysis of the products from *E. coli* strains in single‐strain culture and two‐strain co‐culture, with the addition of substrate **1** and prenol for 36 h. UV absorptions at 280 nm are shown.

We initially conducted *in vitro* cascade reactions with purified AnaPT and YjiC, using naringenin as the acceptor and UDP‐glucose and dimethylallyl diphosphate (DMAPP) as the donors (Figure 1B). High‐Performance Liquid Chromatography (HPLC) and Liquid Chromatography‐High Resolution Mass Spectrometry (LC‐HRMS) analyses of the reaction mixtures indicated the presence of two unknown compounds (**7** and **8**), in addition to the anticipated glucosylated and prenylated intermediates (Figure 1A,B). The synthesis of these compounds was optimized by modifying the reaction sequence. Remarkably, a two‐step process (Reaction I), where prenylation by AnaPT occurred before glycosylation by YjiC, led to a five‐fold increase in the yield of compound **7** and a two‐fold increase in the yield of compound **8**, compared to the opposite order (Reaction II) (Figures 1C and S2). This suggested that YjiC could effectively glycosylate prenylated naringenin, whereas AnaPT showed limited activity toward glycosylated substrates.

Subsequently, a 50 ml enzymatic reaction was conducted to separate compounds **7** and **8**. The two new compounds were purified using semi‐preparative HPLC, and their structures were determined through Nuclear Magnetic Resonance (NMR) spectroscopy (including ^1^H,^13^C, COSY, HSQC, and HMBC) and High‐Resolution Electrospray Ionization Mass Spectrometry (HRESIMS). Compound **7** was assigned the molecular formula C_32_H_40_O_15_ based on HREIMS (*m*/*z* 663.2289 [M‐H]^−^) (Figure S3), suggesting the presence of two glucose units and one isopentenyl group in **7**. The key signals at *δ*_H_ 4.96 (dd, 1H, *J* = 10.4, 7.6), 3.09–3.45 (4H, overlap), and 3.47 (2H, m), and their corresponding carbons individually at *δ*_C_ 99.52 (C‐1″, an anomeric carbon), 76.64 (C‐2″), 77.11 (C‐3″), 69.49 (C‐4″), 73.04 (C‐5″), and 60.57 (C‐6″), combined with the key HMBC correlation (from H‐1″ to C‐7), absolutely proved that the one of the glycosylation positions of naringenin should be at 7‐OH (Table S2 and Figures S4–S8). Another glycosylation site should be at the 4′‐OH position of naringenin based on the key HMBC correlation (from H‐1‴ to C‐4′) (Table S2 and Figures S4–S8). Additionally, the ^1^H‐NMR signals at *δ*_H_ 3.09–3.45 (overlap), 5.31 (m), 1.68 × 2 (s), and ^13^C‐NMR signals at *δ*_C_ 28.12 (C‐1‴), 122.56 (C‐2‴), 131.64 (C‐3‴), 17.75 (C‐4‴), and 25.60 (C‐5‴) showed that there was one prenylation in **7** (Table S2). The prenyl moiety was attached to the C‐3′ position of naringenin based on the key HMBC correlation (from H‐1‴ to C‐2′, C‐3′, and C‐4′) (Figure S8). Thus, compound **7** was identified as 3′‐prenylnaringenin‐4′,7‐*O*‐diglucoside (Figure 1A).

The molecular formula of compound **8** was determined to be C_26_H_30_O_10_ by HREIMS (*m/z* 501.1765 [M‐H]^⁻^). Its NMR data, similar to those of compound **7**, suggested one prenylation and one glycosylation (Figures S5–S9 and S11–S15). The key HMBC correlation from H‐1‴ to C‐4′ proved that the glucose moiety was attached to the 4′‐OH position of naringenin (Figures S9–S10 and S15–S16). Additionally, the ^1^H‐NMR signals also showed one prenylation in compound **8**. The prenyl moiety was also introduced to the C‐3′ position of naringenin through the key HMBC correlation (from H‐1″ to C‐2′, C‐3′, and C‐4′) (Figure S14). The structure of compounds **7** and **8** was definitively established (Figure 1A; Tables S2 and S3), a configuration not previously identified. Consequently, compound **8** was characterized as 3′‐prenylnaringenin‐4′‐*O*‐glucoside (Figure 1A). To the best of our knowledge, both compounds are previously unreported and constitute new additions to the flavonoid family.

We assessed the antimicrobial potential of these two novel compounds. Neither compound demonstrated antibacterial activity against *E. coli*, *Staphylococcus aureus*, or *Bacillus subtilis*. However, compound **7** showed substantial antifungal activity against *Aspergillus niger* (Figure S15), with a minimum inhibitory concentration (MIC) of 400 μg/ml. This activity may be due to the combined effects of enhanced membrane permeability from prenylation and optimized physicochemical properties conferred by glycosylation. In contrast, compound **8** did not display significant antifungal effects under the conditions tested, indicating that the diglycosylated structure in compound **7** might be crucial for its bioactivity.

To establish a sustainable production platform, we engineered *E. coli* BL21 strains for the *de novo* synthesis of novel flavonoids. The *anaPT* gene was codon‐optimized and cloned into pCold‐GST to improve expression. To tackle the limited intracellular supply of DMAPP, we introduced an isopentenol utilization pathway (IUP) through two‐step reactions catalyzed by a promiscuous kinase (PK) and isopentenyl phosphate kinase (IPK) into *E. coli* under two separate T7 promoters. This resulted in a recombinant strain (T2) (Figures 1D and S16), which comprises a promiscuous kinase and isopentenyl phosphate kinase, to enhance DMAPP generation from exogenous prenol^20^.

Initial shake‐flask cultures of strain T2 produced 3′‐prenylnaringenin (**5**) with 1 mM naringenin and 160 mg/l prenol after 36 h (Figure 1F). The co‐expression of *yjiC* and *anaPT*, along with the IUP, enabled the production of compounds **7** and **8** at titers of 47.4 and 6.6 mg/l in the recombinant strain T1 (Figure 1E,F), respectively. We also tested a co‐culture system with two *E. coli* strains, one specializing in prenylation and the other in glycosylation, which resulted in a yield of **7** at 38.0 mg/l and no production of **8.** However, the mono‐culture approach proved more effective for the target compounds (Figure 1F).

In conclusion, we successfully designed and produced two novel naringenin derivatives using a combinatorial biosynthetic approach. By integrating a bacterial glycosyltransferase and a fungal prenyltransferase in a specific catalytic sequence, we generated two non‐natural flavonoids: 3′‐prenylnaringenin‐4′,7‐*O*‐diglucoside (**7**) and 3′‐prenylnaringenin‐4′‐*O*‐glucoside (**8**). Both compounds are unprecedented in nature. Furthermore, we demonstrated the feasibility of producing these compounds in engineered *E. coli* through pathway optimization and precursor engineering. Our work highlights the potential of combining enzymes from diverse biological sources to expand the structural diversity of flavonoids and other natural products. This strategy not only provides access to new bioactive molecules but also offers a sustainable and efficient alternative to traditional extraction or chemical synthesis. Future efforts will focus on optimizing enzyme specificity and reaction conditions, as well as exploring the pharmacological potential of these and other novel flavonoids in greater depth.

## ETHICS STATEMENT

This study did not involve any human participant or animal subject.

## Supporting information

mLife‐2025‐0144.

## Data Availability

All data generated or analyzed in this study are included in this article and additional Supporting Information.
