# Supplementary material for: Creation of bioactive non‐natural flavonoids via combinatorial prenylation–glycosylation cascades
Source: mLife. 2026 Mar 15;5(2):254–8. doi: 10.1002/mlf2.70063 (PMC13131324; doi:10.1002/mlf2.70063)
Supplement: Supplementary file 1 — mLife‐2025‐0144. [file MLF2-5-254-s001.docx]

Creation of bioactive non-natural flavonoids *via* combinatorial prenylation-glycosylation cascades

Hongjiao Zhang^1^, Anxin Zhang^1,2^, Jiatong Ji^1,3^, Wen-Bing Yin^1,2*^

^1^State Key Laboratory of Microbial Diversity and Innovative Utilization, Institute of Microbiology, Chinese Academy of Sciences; Beijing, 100101, China

^2^Medical School, University of Chinese Academy of Sciences; Beijing, 100049, China

^3^Division of Life Sciences and Medicine, University of Science and Technology of China; Hefei, 230027, China

Table of Contents

[Material and Methods 3](#_Toc5794)

[Supplementary Tables 8](#_Toc3371)

[Table S1. (UDP)-glycosyltransferases (UGTs) and prenyltransferases (PTs) used in this study. 8](#_Toc10724)

[Table S2. NMR data of compound 7 (500 MHz for](#_Toc26285) ^[1](#_Toc26285)^[H-NMR and 125 MHz for](#_Toc26285) ^[13](#_Toc26285)^[C-NMR) 9](#_Toc26285)

[Table S3. NMR data of compound 8 (500 MHz for](#_Toc31655) ^[1](#_Toc31655)^[H-NMR and 125 MHz for](#_Toc31655) ^[13](#_Toc31655)^[C-NMR) 10](#_Toc31655)

[Table S4. The plasmids and strains used in this study. 11](#_Toc3622)

[Table S5. The primers used in this study. 12](#_Toc2065)

[Supplementary Figures 13](#_Toc21772)

[Figure S1.](#_Toc4376) *[In vitro](#_Toc4376)* [enzyme reaction of five UDP-dependent glycosyltransferases (UGTs) alone and inactive AnaPT with five UGTs together. 13](#_Toc4376)

[Figure S2. Schematic diagrams of two reaction systems for system optimization of](#_Toc14952) *[in vitro](#_Toc14952)* [cascade reaction. 14](#_Toc14952)

[Figure S3. HRMS spectrum of compound 7. 15](#_Toc4815)

[Figure S4.](#_Toc1670) ^[1](#_Toc1670)^[H-NMR spectrum (500 MHz) of compound 7. 16](#_Toc1670)

[Figure S5.](#_Toc1733) ^[13](#_Toc1733)^[C-NMR spectrum (125 MHz) of compound 7. 17](#_Toc1733)

[Figure S6. HSQC spectrum of compound 7. 18](#_Toc15694)

[Figure S7. COSY spectrum of compound 7. 19](#_Toc22420)

[Figure S8. HMBC spectrum of compound 7. 20](#_Toc25615)

[Figure S9. HRMS spectrum of compound 8. 21](#_Toc11747)

[Figure S10.](#_Toc16139) ^[1](#_Toc16139)^[H-NMR spectrum of (500 MHz) compound 8. 22](#_Toc16139)

[Figure S11.](#_Toc19034) ^[13](#_Toc19034)^[C-NMR spectrum of (125 MHz) compound 8. 23](#_Toc19034)

[Figure S12. HSQC spectrum of compound 8. 24](#_Toc3080)

[Figure S13. COSY spectrum of compound 8. 25](#_Toc31838)

[Figure S14. HMBC spectrum of compound 8. 26](#_Toc10920)

[Figure S15. Antimicrobial activity assay of compounds 7 and 8. 27](#_Toc916)

[Figure S16. Schematic diagram of](#_Toc26723) *[E. coli](#_Toc26723)* [strain expressing](#_Toc26723) *[anaPT](#_Toc26723)* [and IUP (](#_Toc26723)*[EcPK](#_Toc26723)* [and](#_Toc26723) *[AtIPK](#_Toc26723)*[) genes. 28](#_Toc26723)

Material and Methods

Strains and culture conditions

The strains used in this study are listed in Table S4. *Escherichia coli* DH5α for the construction of plasmids and BL21(DE3) for the expression of recombinant proteins and the production of flavonoids were cultivated in LB medium with appropriate antibiotics.

Gene cloning and plasmid construction

Genomic DNA (gDNA) of *E. coli* and *Bacillus licheniformis* was isolated from the cells grown in 3 mL LB at 37 °C for 12 h for the amplification of *yjiC* gene. Total RNAs of *Neosartorya fischeri* and the leaves of *Arabidopsis thaliana* were extracted by the TranZol™ kit (Transgen Biotech, China). The single strand complementary DNA (cDNA) was synthesized by *Evo M-MLV* Plus cDNA Synthesis kit (Accurate Biotech Co. Ltd, China) according to the standard manufacturer's instruction.

*YjiC* and *EcPK* genes were amplified from *B. licheniformis* and *E. coli* gDNA, respectively. *UGT73C6*, *UGT73B1*, and *AtIPK* ORFs were amplified from the cDNA of *Arabidopsis thaliana* wild type leaves. *AnaPT* ORFs were amplified from the cDNA of *Neosartorya fischeri*. *CsUGT75L12* and *CiUGT11* genes were synthesized by Tsingke Biological Technology. TransStart^®^ FastPfu DNA polymerase (TransGen Biotech, China) used to perform PCR reactions. The ORF fragments were inserted into the linearized vectors (*Hind* III-pET28a, *Nco* I/*Nde* I-pCDFDuet, and *Nde* I-pCold-GST) to produce all of the plasmids in this study using the Clone Express^®^ MultiS One Step Cloning Kit (Vazyme Biotech Co. Ltd, China) according to the standard manufacturer's instruction. The plasmids used in this study are listed in Table S4. The oligonucleotide sequences for PCR primers are given in Table S5.

Expression and purification of the recombinant proteins

The expression constructs pYHJ62, pYHJ80, pYHJ188, pYHJ189, pYHJ190, and pYHJ191 were introduced into *E. coli* BL21, respectively. Single colony was cultured in 3 mL LB medium with 50 µg/mL kanamycin at 37 °C overnight. The cells were transferred in 1 L LB media and kept at 37 °C until the optical density (OD_600_) reached 0.6. The recombinant protein was induced with 0.1 mM IPTG at 16 °C for 18 h. Cell pellets were harvested by centrifugation at 4 °C and 5,000 rpm for 10 min, and resuspended in 20 mL lysis buffer (50 mM NaH_2_PO_4_, 300 mM NaCl, 10 mM imidazole, pH 8.0). After disrupting the cells by sonication on ice, the mixtures were centrifuged at 13,000 rpm and 4 °C for 30 min. The supernatant was mixed with Ni-NTA agarose resin for 2 h at 4 °C. The protein-resin mixtures were loaded into a gravity flow column and protein was purified with 20 mM imidazole in buffer (50 mM NaH_2_PO_4_, 300 mM NaCl, pH 8.0). The collected protein solution was desalted with Tris-HCl buffer (50 mM, pH 7.5, 15% glycerol) before being concentrated, aliquoted and flash frozen. Protein concentration was determined on a Nano-Drop C2000 (ThermoFisher Scientific). Purity of the protein was confirmed by SDS-PAGE (Figure S1).

*In vitro* cascade enzymatic reaction of the recombinant proteins

The enzyme assays (50 μL) contained Tris-HCl buffer (50 mM, pH 7.5), CaCl_2_ (10 mM), UDPG (2 mM), DMAPP (2 mM), naringenin (1 mM), the recombinant UGT (10 µM) and the recombinant AnaPT (10 µM). The reactions were incubated at 37 °C for 4 h and terminated with equal volume of methanol. The reaction mixtures were centrifuged at 13,000 rpm for 20 min to remove the protein precipitate before further analysis on high performance liquid chromatography (HPLC) or liquid chromatography mass spectrometry (LC-MS) analysis.

HPLC analysis was conducted with a Waters HPLC system (Waters e2695, Waters 2998, Photodiode Array Detector) using an XTerra MS C18 column (250 by 4.6 mm, 5 μm, Waters). For the secondary metabolites analysis of transformants, water with 0.1% (v/v) formic acid (A) and MeOH (B) was used as the solvent at a flow rate of 1 mL/min. Extracts were eluted with linear gradient from 20 to 100% (v/v) B in 20 min, washed with 100% solvent B for 5 min, and equilibrated with 5% solvent B for 5 min. UV absorption was recorded at 280 nm.

LC-MS analysis was performed on an Agilent HPLC 1200 series system equipped with a single quadrupole mass selective detector and an Agilent 1100LC MSD model G1946D mass spectrometer by using a Venusil XBP C18 column (3.0 by 50 mm, 3 µm, Bonna-Agela Technologies, China). Water (A) with 0.1% (v/v) formic acid and acetonitrile (B) were used as the solvents at a flow rate of 0.5 mL/min. The substances were eluted with a linear gradient from 5 to 100% (v/v) B in 30 min, then washed with 100% solvent B for 5 min, and equilibrated with 5% solvent B for 10 min. The mass spectrometer was set in electrospray positive ion mode for ionization.

Fermentation and metabolite extraction of *E. coli* mutant

For *E. coli* BL21 harboring pYHJ228 and pYHJ229 (T1), a single colony was cultured in LB medium with appropriate antibiotics at 37 ℃ for 12 h. Then the seed was inoculated to 20 mL LB medium at 37 ℃ for about 4 h until OD_600_ reached 0.6. 0.05 mM IPTG and 160 mg/l prenol were added into the culture to induce the expression of *anaPT*, *EcPK* and *AtIPK.* 1 mM naringenin was fed into T1 at 16 °C for 36 h to produce 6-PN and 3'-PN. For *E. coli* BL21 harboring pYHJ62, pYHJ228 and pYHJ229 (T3), the procedures are same as T1. 1 mM naringenin was fed in the mutant at 16 °C for 36 h to produce compounds 7 and 8.

For two-strain co-culture system, T1 was cultured in LB medium with appropriate antibiotics at 37 ℃ for 12 h. Then the seed was inoculated to 10 mL LB medium at 37 ℃ for about 4 h until OD_600_ reached 0.6. 0.05 mM IPTG and 160 mg/l prenol were added into the culture to induce the expression of *anaPT*, *EcPK* and *AtIPK.* 1 mM naringenin was fed in the mutant at 16 °C for 18 h to produce 6-PN and 3'-PN. Subsequently, 10 mL T2 cultured in LB medium with 0.05 mM IPTG at 16 °C for 18 h was added in the culture of T1 for another 18 h fermentation at 16 °C.

The 36-h culture was extracted thrice with ethyl acetate (EtOAc). The extracts were evaporated and dissolved in 200 μL methanol (MeOH) and centrifuged at 13,000 rpm for 20 min before further analysis on HPLC.

Quantification of flavonoids in *E. coli* mutant

For quantification of flavonoids produced by engineered *E. coli* strain, three independent single colonies, with the relevant genetic modifications, were inoculated into LB medium with appropriate antibiotics at 37 ℃ for 12 h. Next, the seeds were inoculated into 20 mL of LB medium and cultured at 37 °C until OD_600_ reached 0.6. 0.05 mM IPTG, 160 mg/l prenol and 1 mM naringenin were added into the culture at 16 °C for 36 h. Cell culture was extracted thrice with EtOAc and the organic phase was then concentrated under reduced pressure, dissolved in 200 μL MeOH and 10 μL sample was injected for HPLC quantitative analysis.

Product purification and structure characterization

To isolate 7 and 8, a 50-mL *in vitro* cascade enzymatic reaction was performed at 37 °C for 4 h. Equal volume of methanol was used for extraction five times and the organic phase was then concentrated under reduced pressure, dissolved in 2 mL MeOH for the purification of compounds 7 and 8. The target compounds 7 and 8 were purified by semi-preparative HPLC (ACN: H_2_O, 30:70).

Semipreparative purification on HPLC was performed on an SSI HPLC system (Teledyne SSI Lab Alliance Series III pump system and Series 1500 Photodiode Array Detector) with an ODS column (C18, 10.0 by 250 mm, 5 μm, YMC) and a flow rate of 2 mL/min.

NMR spectra (^1^H, ^13^C) were recorded on a Bruker Avance-500 MHz spectrometer using TMS as internal standard (Bruker Corporation, Karlsruhe, Germany). All spectra were processed with MestReNova 14.2.1 (Metrelab). Chemical shifts are referenced to those of the solvent signals.

Antimicrobial activity of assay

For compound and strain preparation, ampicillin, naringenin, compounds 7 and 8 were dissolved in dimethyl sulfoxide (DMSO), respectively. The following bacteria were used in this study: *Escherichia coli*, *Staphylococcus aureus* and *Bacillus subtilis*. LB medium mixed with bacterial cells (1 × 10^8^ cells) were plated and sterile filter papers with 10 μL compound (1 mg/mL) were placed on the plate for the culture at 37 °C for 12 h. The following fungal strains were used in this study: *Aspergillus flavus*, *A. niger*, and *A. fumigatus*. Fungal strains were cultured in potato dextrose agar (PDA) medium at 37 °C or 30 °C (*A. flavus*) until the diameter reaches 2 cm. Sterile filter papers with 10 μL compound (3 mg/mL) were placed 1 cm from the edge of the colony for the culture at 37 °C or 30 °C (*A. flavus*) until the mycelium covered the filter paper with DMSO. To determine the MIC value of compound 7, *A. niger* cells (2 × 10^3^ cells) were plated into 96-microwell plates with RPMI1640 medium and compounds (0.2 mL/well) for the culture at 37 °C for 2 days.

## Supplementary Tables

## Table S1. (UDP)-glycosyltransferases (UGTs) and prenyltransferases (PTs) used in this study.

| UGTs/PTs | Strain | Position | Efficiency |
| --- | --- | --- | --- |
| YjiC | *Bacillus licheniformis* | C4’-OH、C7-OH | 80% |
| UGT73C6 | *Arabidopsis thaliana* | C7-OH | 70% |
| CsUGT75L12 | *Camellia sinensis* | C7-OH | 15% |
| CiUGT11 | *Chrysanthemum indicum* | C7-OH | 40% |
| UGT73B1 | *Arabidopsis thaliana* | C7-OH | 80% |
| 7-DMATS | *Aspergillus fumigatus* | C-6、C-3' | 29.6% |
| AnaPT | *Neosartorya fischeri* | C-6、C-3' | 54.2% |

##
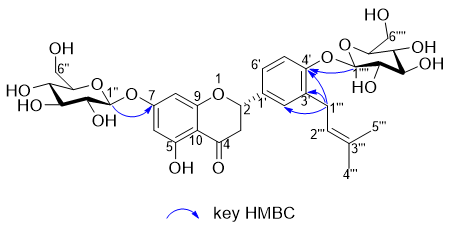
Table S2. NMR data of compound 7 (500 MHz for ^1^H-NMR and 125 MHz for ^13^C-NMR)

| Position | *δ*_C_ (type) | *δ*_H_ (type, *J* in Hz) | Key HMBC |
| --- | --- | --- | --- |
| 1 | - | - |  |
| 2  3  4  5  6  7  8  9  10  1'  2'  3'  4'  5'  6'  1''  2''  3''  4''  5''  6''  1'''  2'''  3'''  4'''  5'''  1''''  2''''  3''''  4''''  5''''  6'''' | 78.57, CH  42.09, CH_2_  197.08, C  165.26, C  96.59, CH  165.35, C  95.47, CH  162.65, C  103.31, C  131.53, C  127.93, CH  130.44, C  155.51, C  114.87, CH  125.70, CH  99.52, CH  76.34, CH  77.11, CH  69.49, CH  73.04, CH  60.57, CH_2_  28.12, CH_2_  122.56, CH  131.64, C  17.75, CH_3_  25.60, CH_3_  101.12, CH  76.78, CH  77.11, CH  69.80, CH  73.43, CH  60.79, CH_2_ | 5.54 dt (14.9, 8.4)  3.09-3.45 overlap  -  -  6.13 dd (4.9, 2.2)  -  6.16 d (2.2)  -  -  -  7.26 s  -  -  7.11 d (8.5)  7.29 dt (8.5, 2.2)  4.96 dd (10.4, 7.6)  3.09-3.45 overlap  3.09-3.45 overlap  3.09-3.45 overlap  3.09-3.45 overlap  3.47 m  3.09-3.45 overlap  5.31, m  -  1.68 s  1.68 s  4.82 m  3.09-3.45 overlap  3.09-3.45 overlap  3.09-3.45 overlap  3.09-3.45 overlap  3.69 m | C-7  C-2', 3', 4'  C-4' |

NMR data for compound 7 was recorded in DMSO-*d*_6_.

## Table S3. NMR data of compound 8 (500 MHz for ^1^H-NMR and 125 MHz for ^13^C-NMR)


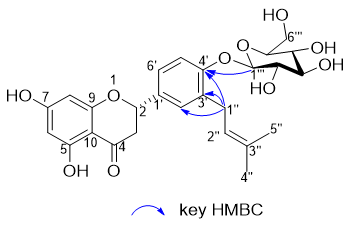


| Position | *δ*_C_ (type) | *δ*_H_ (type, *J* in Hz) | Key HMBC |
| --- | --- | --- | --- |
| 1 | - | - |  |
| 2  3  4  5  6  7  8  9  10  1'  2'  3'  4'  5'  6'  1''  2''  3''  4''  5''  1'''  2'''  3'''  4'''  5'''  6''' | 80.21, CH  44.00, CH_2_  197.45, C  165.43, C  97.12, CH  168.60, C  96.28, CH  164.67, C  103.38, C  133.99, C  128.60, CH  133.58, C  156.89, C  116.13, CH  126.22, CH  29.38, CH_2_  123.65, CH  132.65, C  17.95, CH_3_  25.93, CH_3_  102.49, CH  78.19, CH  78.30, CH  71.39, CH  75.03, CH  62.56, CH_2_ | 5.41 d (11.6)  3.08 s  2.77 m  -  -  5.90 d (1.9)  -  5.92 m  -  -  -  7.25 s  -  -  7.18 d (8.5)  7.29 d (8.5)  3.38-3.56 overlap  5.36 dt (7.5, 5.2)  -  1.74 s  1.76 s  4.96 d (7.3)  3.38-3.56 overlap  3.38-3.56 overlap  3.38-3.56 overlap  3.38-3.56 overlap  3.92 dd (12.0, 2.0)  3.72 dd (12.0, 5.2) | C-2', 3', 4'  C-4' |

NMR data for compound 8 was recorded in CD_3_OD.

## Table S4. The plasmids and strains used in this study.

| Plasmids/ Strains | Description | Reference |
| --- | --- | --- |
| pET28a | Kan^r^, T7 promoter, lacI | Lab stock |
| pCold-GST | Amp^r^, cspA promoter, pBR322 origin of replication | Lab stock |
| pCDFDuet | Cm^r^, T7 promoter, p15A origin of replication | Lab stock |
| pYHJ62 | *YjiC* ORF in pET28a | This study |
| pYHJ80 | *AnaPT* ORF in pET28a | This study |
| pYHJ188 | *UGT73C6* ORF in pET28a | This study |
| pYHJ189 | *CsUGT75L12* ORF in pET28a | This study |
| pYHJ190 | *CiUGT11* ORF in pET28a | This study |
| pYHJ191 | *UGT73B1* ORF in pET28a | This study |
| pYHJ228 | *EcPK* ORF-*AtIPK* ORF in pCDFDuet | This study |
| pYHJ229 | *AnaPT* ORF in pCold-GST | This study |
| *Escherichia coli* DH5α | *Escherichia coli* | Lab stock |
| *Escherichia coli* BL21 (DE3) | *Escherichia coli, initial strain* | Lab stock |
| *Escherichia coli* | *Escherichia coli* | Lab stock |
| *Glucococcus aureus* | *Glucococcus aureus* | Lab stock |
| *Bacillus subtilis* | *Bacillus subtilis* | Lab stock |
| *Aspergillus flavus* | *Aspergillus flavus* | Lab stock |
| *Aspergillus niger* | *Aspergillus niger* | Lab stock |
| *Aspergillus fumigatus* | *Aspergillus fumigatus* | Lab stock |
| T1 | *E. coli* BL21 (DE3) harboring pYHJ228 and pYHJ229 | This study |
| T2 | *E. coli* BL21 (DE3) harboring pYHJ62 | This study |
| T3 | *E. coli* BL21 (DE3) harboring pYHJ62, pYHJ228 and pYHJ229 | This study |

## Table S5. The primers used in this study.

| Primers | Oligonucleotide sequences (5’- 3’) | Description |
| --- | --- | --- |
| pYHJ62-1-F | tgactggtggacagcaaatgggtcgcatgggacataaacatatcgcgat | Amplification of fragment 1 for pYHJ62 |
| pYHJ62-1-R | tctcagtggtggtggtggtggtgctcgagttttactcctgcgggtgcta |  |
| pYHJ80-1-F | tgactggtggacagcaaatgggtcgcatgtctcccttgtctatgcaaac | Amplification of fragment 1 for pYHJ80 |
| pYHJ80-1-R | tctcagtggtggtggtggtggtgctcgaggagattgcccttcataccac |  |
| pYHJ188-1-F | gtggacagcaaatgggtcgcatggctttcgaaaaaaacaacga | Amplification of fragment 1 for pYHJ188 |
| pYHJ188-1-R | gctcgagtgcggccgcaagcttattattggactgtgctagttgca |  |
| pYHJ189-1-F | gtggacagcaaatgggtcgcatggtgcaacacggacac | Amplification of fragment 1 for pYHJ189 |
| pYHJ189-1-R | tcgagtgcggccgcaagcttgaggcaatcaccaccgac |  |
| pYHJ190-1-F | agccatatggctagcatgactggtggacagcaaatgggtcgcatggactccgcggcgac | Amplification of fragment 1 for pYHJ190 |
| pYHJ190-1-R | gtggtggtggtggtgctcgagtgcggccgcaagcttgttgaagttgttagtgccggtcc |  |
| pYHJ191-1-F | gtggacagcaaatgggtcgcatgggaactcctgtcgaag | Amplification of fragment 1 for pYHJ191 |
| pYHJ191-1-R | tcgagtgcggccgcaagctttaccttctctttttgcagtttaactaac |  |
| pYHJ228-2-F | ctttaataaggagatataccatgcaagtcgacctgctg | Amplification of fragment 1 for pYHJ228 |
| pYHJ228-2-R | tgatggtgatggctgctgcctgcctgcacctcctgcgt |  |
| pYHJ228-3-F | ggcagcagccatcaccat | Amplification of fragment 2 for pYHJ228 |
| pYHJ228-3-R | atgtatatctccttcttatacttaactaat |  |
| pYHJ228-4-F | tataagaaggagatatacatatggaactgaacatctctgaatc | Amplification of fragment 3 for pYHJ228 |
| pYHJ228-4-R | gatatccaattgagatctgccttggagaaacggatgatggtac |  |
| pYHJ229-2-F | ccctcgagggtaccgagctctcagagattgcccttcataccacc | Amplification of fragment 1 for pYHJ229 |
| pYHJ229-2-R | aagttctgttccagggcccgatgtctcccttgtctatgcaaac |  |

## Supplementary Figures


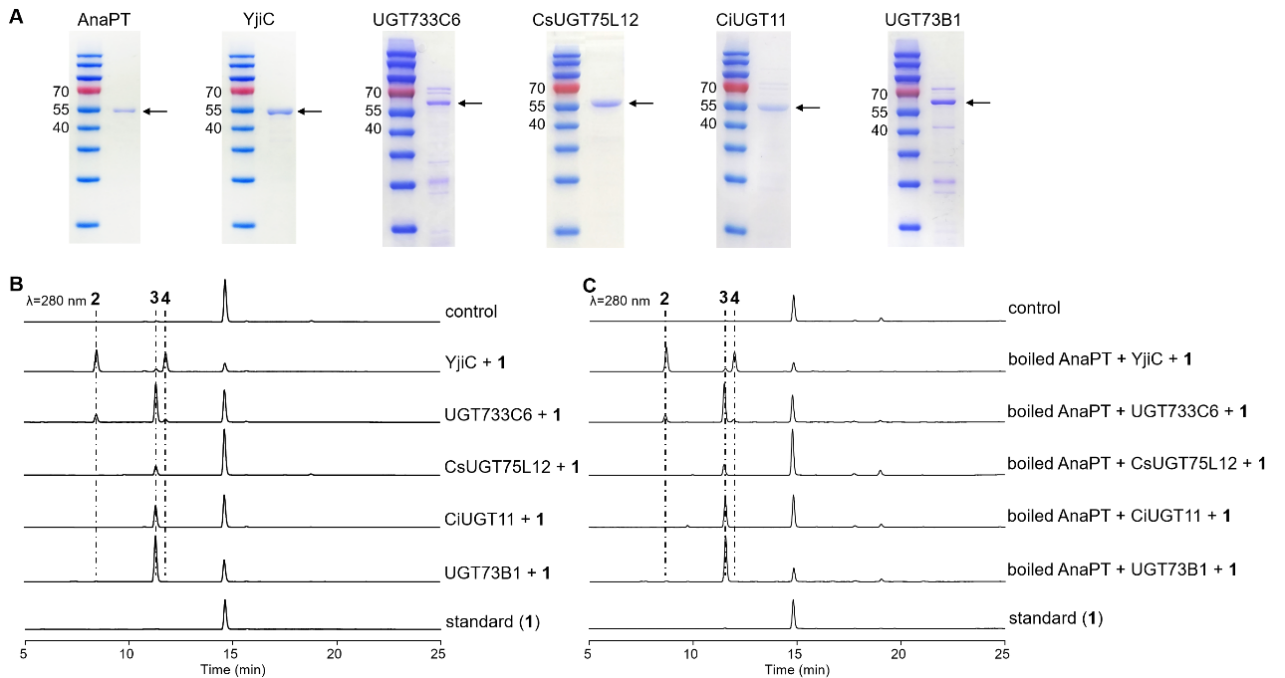


## Figure S1. *In vitro* enzyme reaction of five UDP-dependent glycosyltransferases (UGTs) alone and inactive AnaPT with five UGTs together.

(A) SDS-PAGE analysis of AnaPT and five UGTs. The black arrow indicates the target protein band. AnaPT (53.3 kDa), YjiC (49.1 kDa), UGT733C6 (60.8 kDa), CsUGT75L12 (57.4 kDa), CiUGT11 (56.6 kDa) and UGT73B1 (59.8 kDa). (B) HPLC analysis of the enzymatic reaction of five UGTs with 1. (C) HPLC analysis of the enzymatic reaction of five UGTs and inactive AnaPT with 1. 2, naringenin 4',7-*O*-diglucoside; 3, naringenin 7-*O*-glucoside; 4, naringenin 4’-*O*-glucoside. UV absorptions at 280 nm are illustrated.


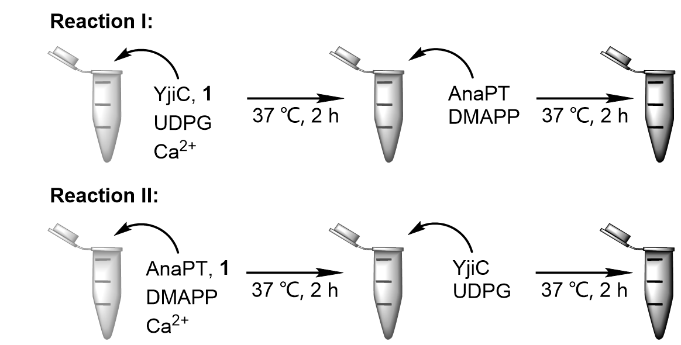


## Figure S2. Schematic diagrams of two reaction systems for system optimization of *in vitro* cascade reaction.

## Figure S3. HRMS spectrum of compound 7.

## Figure S4. ^1^H-NMR spectrum (500 MHz) of compound 7.

## Figure S5. ^13^C-NMR spectrum (125 MHz) of compound 7.

x2

## Figure S6. HSQC spectrum of compound 7.

## Figure S7. COSY spectrum of compound 7.

## Figure S8. HMBC spectrum of compound 7.

Figure S9. HRMS spectrum of compound 8.

## Figure S10. ^1^H-NMR spectrum of (500 MHz) compound 8.

## Figure S11. ^13^C-NMR spectrum of (125 MHz) compound 8.

## Figure S12. HSQC spectrum of compound 8.

## Figure S13. COSY spectrum of compound 8.

## Figure S14. HMBC spectrum of compound 8.

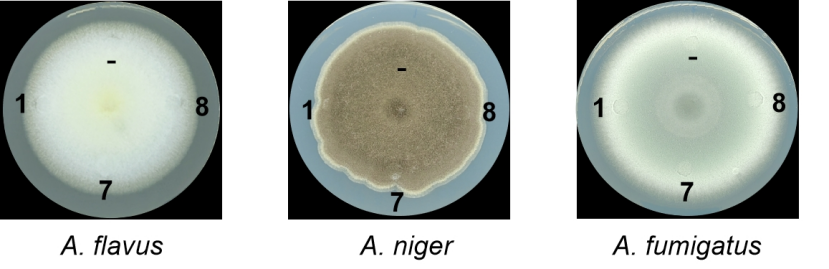


## Figure S15. Antimicrobial activity assay of compounds 7 and 8.

“-” represents negative control (DMSO).


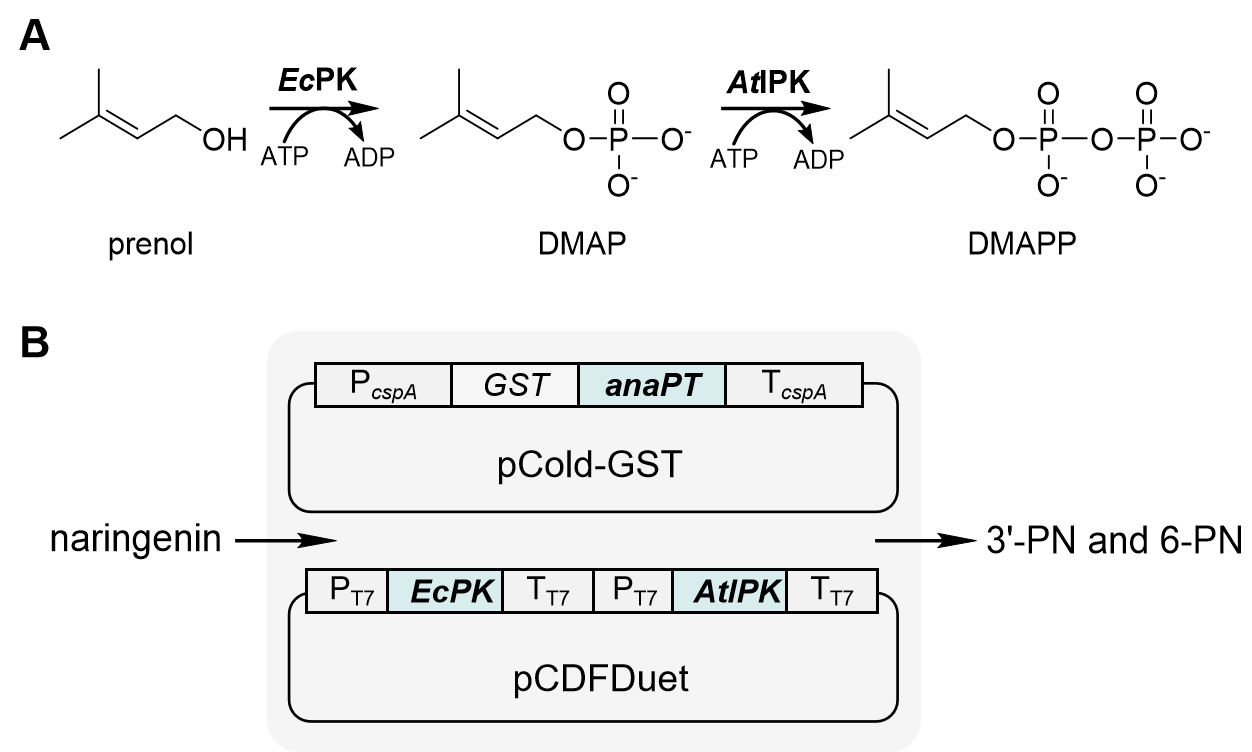


Figure S16. Schematic diagram of *E. coli* strain expressing *anaPT* and IUP (*EcPK* and *AtIPK*) genes.

(A) The isopentenol utilization pathway (IUP) can produce the basic isoprenoid metabolic intermediates DMAPP in two steps using prenol as feedstock. (B) *E. coli* strain expressing *anaPT* and IUP (*EcPK* and *AtIPK*) genes produces 3’-PN and 6-PN using naringenin as the substrate.
